# Lysosomal Ca^2+^ Signaling Regulates High Glucose-Mediated Interleukin-1β Secretion *via* Transcription Factor EB in Human Monocytic Cells

**DOI:** 10.3389/fimmu.2017.01161

**Published:** 2017-09-15

**Authors:** Hisa Hui Ling Tseng, Chi Teng Vong, Yiu Wa Kwan, Simon Ming-Yuen Lee, Maggie Pui Man Hoi

**Affiliations:** ^1^State Key Laboratory of Quality Research in Chinese Medicine, Institute of Chinese Medical Sciences, University of Macau, Taipa, Macau; ^2^Faculty of Medicine, School of Biomedical Sciences, The Chinese University of Hong Kong, Shatin, Hong Kong

**Keywords:** high glucose, lysosomal Ca^2+^, Ca^2+^ homeostasis, lysosomal exocytosis, interleukin-1β, monocytes

## Abstract

Aberrant activation of the innate immune system, including NOD-like receptor pyrin domain containing 3 (NLRP3) inflammasome-dependent interleukin-1β (IL-1β) secretion, has been implicated in the pathogenesis of type 2 diabetes mellitus (T2DM) and its complication. Our previous study demonstrated that hyperglycemia, a hallmark characteristic of T2DM, induced NLRP3 inflammasome-dependent caspase-1 activation and IL-1β maturation in human monocytic cells. In this study, we examined the underlying mechanisms of secreting IL-1β during hyperglycemia, with a focus on the alteration of Ca^2+^ homeostasis and lysosomal exocytosis. We found that high glucose (HG; 30 mM glucose for 48 h) altered Ca^2+^ homeostasis by reducing lysosomal Ca^2+^ concentration that appeared to be resulted from Ca^2+^ moving out of lysosomes into cytosol in human monocytic cell lines, U937 and THP-1 cells. Moreover, HG-induced lysosomal Ca^2+^-dependent mature IL-1β release was strongly correlated with the activation and upregulation of two lysosomal marker proteins, cathepsin D and lysosomal-associated membrane protein-1 (LAMP-1). This involved calcineurin/transcription factor EB (TFEB) pathway and its target genes, cathepsin B, cathepsin D, and LAMP-1, to mediate lysosomal exocytosis. Therefore in this study, we revealed a novel mechanism of HG-induced lysosomal exocytosis which was regulated by lysosomal Ca^2+^ signals through calcineurin/TFEB pathway, thus contributing to IL-1β secretion in human monocytic cells.

## Introduction

Interleukin-1β (IL-1β) is one of the pro-inflammatory cytokines that is involved in the pathogenesis of type 1 diabetes, type 2 diabetes mellitus (T2DM), and diabetic vascular complication, such as atherosclerosis (1–3). IL-1β mediates inflammatory responses contributing to impaired insulin secretion and sensitivity in insulin-sensitive cells (2, 4). Indeed, IL-1β maturation was tightly controlled by the inflammasome, a multiprotein complex that consists of an inflammasome sensor molecule, the adaptor protein apoptosis-associated speck-like protein containing a C-terminal caspase recruitment domain (ASC) and caspase-1 (5). NOD-like receptor pyrin domain containing 3 (NLRP3) inflammasome is now the best studied inflammasome and has been implicated in the progression of T2DM (6, 7). Recent studies suggested that the activation of NLRP3 inflammasome was a key mechanism in obesity- and high-fat diet-induced insulin resistance and inflammation (8, 9). Moreover, our previous study demonstrated that hyperglycemia, a hallmark of T2DM, could induce reactive oxygen species (ROS)-sensitive NLRP3 inflammasome activation in human monocytes (10), suggesting that high glucose (HG) is a key factor of activated innate immunity in T2DM, which could be sensed by NLRP3 inflammasome and mediate the processing of IL-1β under diabetic condition.

It has been recognized that there are three steps involved in IL-1β secretion, first step is to stimulate the synthesis of pro-IL-1β, then pro-IL-1β is cleaved into mature IL-1β by caspase-1, which is followed by IL-1β secretion *via* non-classical secretory pathway into the extracellular milieu (11). In most phagocytic cells, such as monocytes, macrophages, and dendritic cells, IL-1β secretion was associated with the exocytosis of secretory lysosomes (11), which suggested the importance of lysosomes in IL-1β secretory pathways. Indeed, conventional lysosome is defined by the common function of degrading or recycling processes of intracellular materials (12). In monocytes or macrophages, lysosomes also serve as a secretory compartment for sorting and secretory pathways (13). There are two key features of secretory lysosomal exocytosis. First, signals stimulate the recruitment of lysosomes trafficking to the plasma membrane (PM). Second, intracellular Ca^2+^ concentration ([Ca^2+^]_i_) rise triggers secretory lysosomes to fuse with the PM and release secretory proteins (13, 14). Ca^2+^ influx was known to be a critical regulator of lysosomal exocytosis to mediate IL-1β secretion (15, 16), and prolonged hyperglycemia was known to be resulted in Ca^2+^ influx and an increase in [Ca^2+^]_i_ in different cell types (10, 17–19). Moreover, our previous study has demonstrated that HG could enhance [Ca^2+^]_i_ and induced caspase-1-dependent IL-1β secretion *via* transient receptor potential melastatin-2 (TRPM2) in human monocytic cells (10). However, the mechanism of secreting IL-1β into extracellular milieu by HG remains to be clarified.

Transcription factor EB (TFEB) is an essential transcriptional regulator for lysosomal function (20, 21), which was regulated by lysosomal Ca^2+^ signals that could promote cellular processes, including autophagy and lysosomal exocytosis (22–24). Furthermore, lysosomal Ca^2+^ release by glycyl-l-phenylalaninebeta-naphthylamide (GPN) could cooperate with endoplasmic reticulum (ER) Ca^2+^ store and resulted in lysosomal exocytosis and IL-1β secretion in human monocytic cells (15, 25). These observations indicated a close relationship between the alteration of Ca^2+^ homeostasis and lysosomal exocytosis. Although many stimuli were shown to activate TFEB and mediate lysosome-dependent cellular processes (26), it is unclear how TFEB mediates these processes at the transcriptional level.

Here, we used hyperglycemic environment to mimic the diabetic condition *in vitro*. Treatment with 30 mM glucose for 48 h was regarded as the HG model in U937 and THP-1 monocytic cells. In this study, we demonstrated that HG could induce change in [Ca^2+^]_i_ and affect lysosomal Ca^2+^ homeostasis, and mediate lysosomal exocytosis. We also found that this lysosomal Ca^2+^ signaling by HG could trigger calcineurin/TFEB pathway and its target genes cathepsin D and lysosomal-associated membrane protein-1 (LAMP-1), and then subsequently release IL-1β in human monocytic cells.

## Materials and Methods

### Reagents and Chemicals

Carbonyl cyanide 3-chlorophenylhydrazone (CCCP), ethylene glycol tetra acetic acid (EGTA), hydrogen peroxide solution (H_2_O_2_), d-mannitol, and lipopolysaccharides were purchased from Sigma-Aldrich, USA. Bafilomycin A1 and GPN were from Santa Cruz Biotechnology, while BAPTA-AM, cyclosporin A, FK506, ionomycin, nicotinic acid adenine dinucleotide phosphate (NAADP), *trans*-Ned-19 (Ned-19), and U18666A were from Tocris Biosciences, USA. Thapsigargin (TG) was bought from Almone Labs, USA, while LysoTracker Red DND-99 Dye and Rhod dextran were from Invitrogen, USA. Antibodies used for immunoblotting and immunostaining were as follows: anti-mouse lysosome-associated membrane protein-1 (LAMP-1; sc-20011, Santa Cruz Biotechnology, USA), anti-rabbit cathepsin D (2284S, Cell Signaling, USA), anti-rabbit caspase-1 (2225S, Cell Signaling, USA), anti-rabbit TFEB (37785S, Cell Signaling, USA), anti-rabbit histone H3 (D1H2) (4499S, Cell Signaling, USA), anti-rabbit Integrin β1 (4706S, Cell Signaling, USA), anti-rabbit GAPDH (2118S, Cell Signaling, USA), and anti-rabbit α/β-tubulin (2148S, Cell Signaling, USA).

### Cell Culture, Treatments, and ELISA

U937 (ATCC, USA) and THP-1 (InvivoGen, USA) monocytic cell lines were grown in RPMI 1640 (Gibco, USA) supplemented with 10% FBS, 2 mM l-glutamine, and 100 U/mL of penicillin and streptomycin. In HG experiments, before HG stimulation, the cells were cultured in RPMI 1640 with 5.5 mM glucose for 48 h, and then were changed to 10, 20, or 30 mM glucose RPMI 1640 for indicated time points. 30 mM mannitol was used as an osmotic control. For the experiments using chemical inhibitors, Cs A, FK506, and U18666A were pre-treated for 24 h, while TG was pre-treated for 45 min. EGTA and BAPTA-AM were treated in the presence of HG stimulation. For the immunoblotting experiments measuring TFEB translocation by calcium inducers, GPN, H_2_O_2_, ionomycin, NAADP, and TG were stimulated for 20 min. The supernatants from U937 and THP-1 cells were collected for the detection of human IL-1β levels by ELISA (eBioscience, USA).

### Specific Small Interfering RNA (siRNA) Experiments

Cells were transiently transfected with TFEB siRNA (100 nmol/L; Ambion, USA), by using Lipofectamine^®^ RNAiMAX Transfection reagent (Gibco, USA). The protocol was synthesized according to the manufacturer’s protocol. GAPDH siRNA was used as a control (40 nmol/L; Ambion, USA). Transfection efficiency was >70% assessed by BLOCK-iT™ Alexa Fluor^®^ Red Fluorescent Control (Ambion, USA) and western blotting. Cells were transfected with siRNA for 24 h before experiments.

### [Ca^2+^]_i_ Measurements

The intracellular Ca^2+^ concentration ([Ca^2+^]_i_) was measured in single cells as previously described (27). Cells were loaded with Fluo-4 AM (2 µM; Molecular Probes, USA) in Tyrode solution containing 136.5 mM NaCl, 5.4 mM KCl, 0.53 mM MgCl_2_, 1.8 mM CaCl_2_, 0.33 mM NaH_2_PO_4_, 5.5 mM glucose, and 5.5 mM HEPES (pH 7.4, adjusted with NaOH) for 30 min at 37°C. Fluo-4 fluorescence intensity (494 nm excitation; 506 nm emission) was sampled at 5 s intervals using a Cell^®^ system (MT20, Olympus, USA). To enable comparisons between cells, the maximal change in fluorescence intensity was measured before and after GPN (400 µM), NAADP (1 µM), Baf A1 (500 nM), or TG (1 µM) was added.

### Lysosomal Ca^2+^ Measurements

The lysosomal Ca^2+^ concentration was measured as previously described (28). For lysosomal Ca^2+^ measurements, the cells were incubated with Rhod dextran (25 mg/ml) for 12 h after treatment as indicated in results, while for all cytosolic Ca^2+^ measurements, the cells were incubated with Fluo-4 (2 µM) for 30 min. The median fluorescence intensity (MFI) was determined using a FACS Canto flow cytometer (BD Biosciences, USA), and the data were analyzed using FlowJo software (Tree Star, USA).

### Western Blot Analysis

Total protein was extracted with ice-cold lysis buffer, the nuclear/cytosolic proteins were extracted by using the Nuclear and Cytoplasmic Extraction Kit (Pierce, USA), and the PM/cytosolic proteins were extracted by using the Mem-PER™ Plus Membrane Protein Extraction Kit (Pierce, USA). The protein concentrations of the lysates were measured by the bicinchoninic acid kit (Pierce, USA). 40 µg proteins were used and separated by 10% SDS-PAGE gels and were transferred onto the nitrocellulose membranes. Membranes were incubated with primary antibodies (1/1,000 dilution) overnight at 4°C, and secondary antibodies (1/1,000 dilution) for 1 h at room temperature, and the immunoblots were developed by enhanced chemiluminescence (GE Healthcare Life Sciences, USA) with a ChemiDoc™ MP System (Bio-Rad Laboratories, USA). GAPDH, β-actin, α/β-Tubulin, Histone H3, and Integrin β1 were used as housekeeping controls.

### Real-time PCR Analysis

Total RNA was extracted using RNeasy Mini Kit (Qiagen, USA), and cDNA was synthesized using High-Capacity cDNA Reverse Transcription Kit (Applied Biosystems, USA). cDNA was quantified using Taqman assays by ViiA 7 Real-Time PCR System (Applied Biosystems, USA). The Taqman probes (Applied Biosystems, USA) used were as follows: TFEB (Hs00292981_m1), Cathepsin B (Hs00947433_m1), Cathepsin D (Hs00157205_m1), LAMP-1 (Hs00174766_m1), IL-1β (Hs00174097_m1), and β-actin (4326315E). β-Actin was used as an endogenous control. All gene expressions were calculated using the ΔΔCt method and were normalized to control.

### Flow Cytometry

For cells labeling with lysotracker, the cells were incubated with LysoTracker DND-99 Dye (250 nM) for 45 min at 37°C after treatment as indicated in results. The MFI was determined using a FACS Canto flow cytometer (BD Biosciences, USA), and the data were analyzed using FlowJo software (Tree Star, USA).

The LAMP-1 level on the PM was measured as previously described (29). After treatment as indicated in results, the intact cells were incubated with LAMP-1 antibody overnight at 4°C and then fixed with 4% paraformaldehyde solution (PFA; Santa Cruz Biotechnology, USA). After fixation, the cells were incubated with secondary antibody (1/400 dilution). The MFI was determined using a FACS Canto flow cytometer (BD Biosciences, USA), and the data were analyzed using FlowJo software (Tree Star, USA).

### β-Hexosaminidase Secretion Assay

β-Hexosaminidase secretion was measured as previously described (25). After treatment, 200 ml supernatants of the cells were equilibrated in 1 mM EGTA-Ca^2+^-free buffer for 3 h and then mixed with 200 ml of 1 mM 4-methylumbelliferyl *N*-acetyl-β-d-glucosaminide (Sigma-Aldrich, USA) in 0.1 M citrate buffer (0.05 M citric acid, 0.05 M sodium citrate, pH 4.5, Sigma-Aldrich, USA) for 1 h at 37°C. The reaction was stopped with 400 ml 0.1 M sodium carbonate buffer (Sigma-Aldrich, USA), and the absorbance was measured at 405 nm. To determine the total cellular content of β-hexosaminidase, the cells were lysed with 1% (v/v) Triton X-100, and 10 µl of the cell extracts were used for the enzyme activity reaction. The percentage of β-hexosaminidase release was calculated from the enzyme activity of the supernatants and lysates.

### Cathepsin D Activity Assay

Cells were extracted with 200 µl of chilled Cell Lysis Buffer following the manufacturer’s instruction. Cathepsin D activity was measured by using a flourimetric assay kit (Abcam, USA) and was normalized to control.

### TFEB Nuclear Translocation Assay

After treatment as indicated in results, the cells were fixed with 4% PFA for 15 min, followed by permeabilization with 0.1% Triton X-100 for 5 min, and were blocked in 20% goat serum (Cell Signaling, USA) for 30 min. Next, the cells were incubated with TFEB antibody (1/50 dilution) overnight at 4°C, and stained with secondary antibody (1/400 dilution) for 1 h and DAPI for 10 min. For the acquisition of the images, at least six images were taken per well of the 96-well plate by IN Cell Analyzer 2000 (GE Healthcare, USA), and quantitative analysis was performed with ImageJ software.

### Immunofluorescence Staining

The cells were seeded onto confocal dishes (SPL Life Sciences, Korea) and were treated with indicated conditions as described. The cells were fixed with 4% PFA for 15 min, blocked in 20% goat serum (Cell Signaling, USA) for 30 min, and incubated with primary antibodies (1/50 dilution) overnight at 4°C, and then secondary antibodies (1/400 dilution) for 1 h. Images were captured with a confocal microscope (LEICA TCS SP8, Leica Microsystems, Germany), and quantitative analysis was performed with the ImageJ software.

### Statistical Analysis

All data were expressed as mean ± SEM and were analyzed by GraphPad Prism 5.0 (GraphPad, USA). Significant differences were determined by one-way ANOVA followed by a Dunnett’s test. *P* < 0.05 was considered as significant. Sample size (*n*) represented the number of independent experiments.

## Results

### HG Alters Lysosomal Ca^2+^ Homeostasis in Human Monocytic Cells

**Figure 1:**
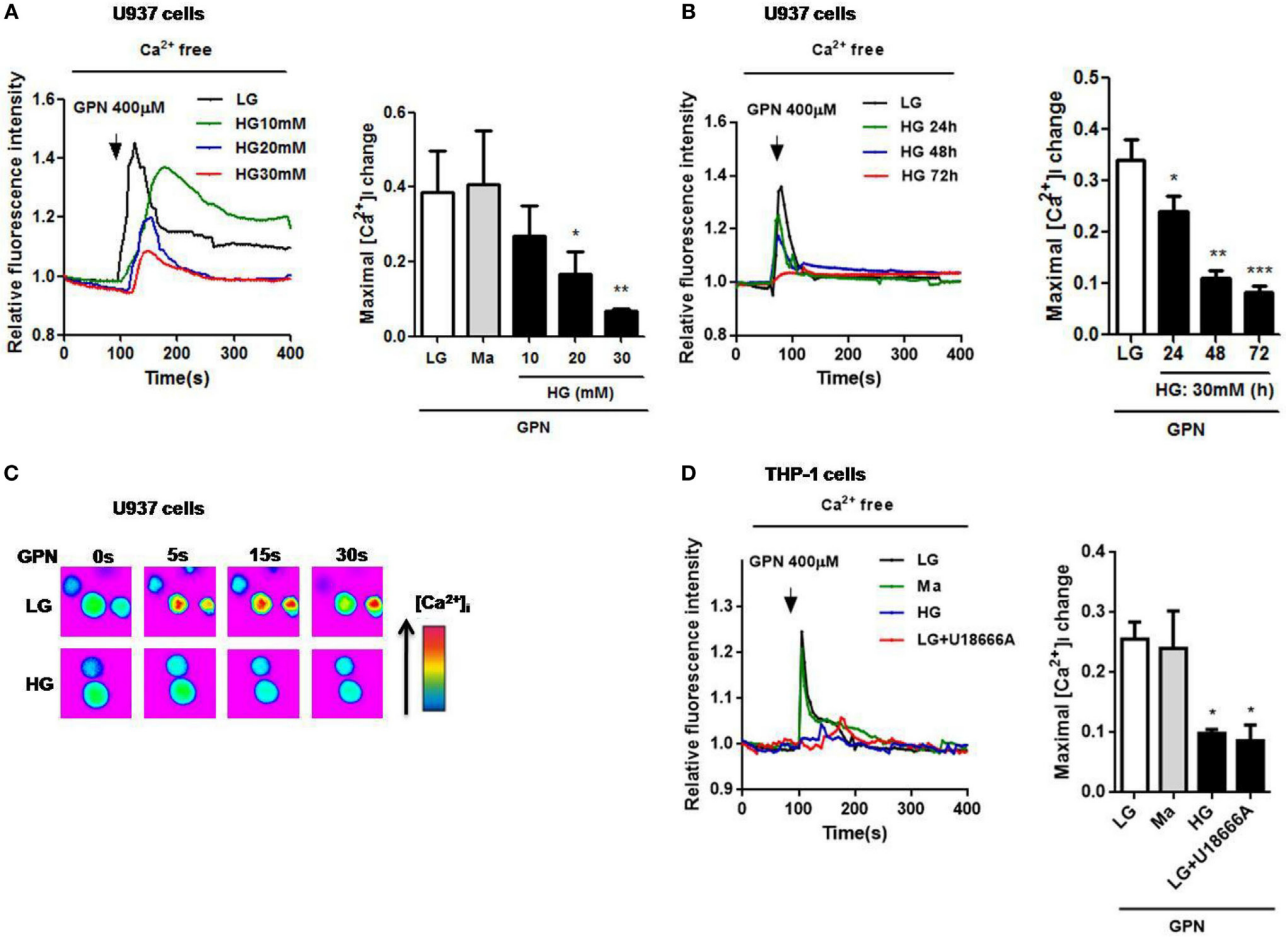
High glucose (HG) reduced GPN-evoked lysosomal Ca^2+^ release in U937 and THP-1 cells. The cells were loaded with Fluo-4-AM and were treated with glycyl-l-phenylalanine-beta-naphthylamide (GPN) to evoke Ca^2+^ responses. Representative and relative changes in intracellular Ca^2+^ concentration ([Ca^2+^]_i_) evoked by GPN (400 µM) under low glucose (LG; 5.5 mM glucose), mannitol (Ma; 30 mM mannitol) or **(A)** HG (10, 20, 30 mM glucose for 48 h), or **(B)** HG (30 mM glucose) for 24, 48, 72 h (*n* = 4–5), or **(C)** HG (30 mM glucose for 48 h) in U937 cells. **(D)** Representative and relative changes in [Ca^2+^]_i_ evoked by GPN (400 µM), with or without pre-treatment of U18666A (2 µg/ml) under HG (30 mM glucose for 48 h) in THP-1 cells (*n* = 4). Data were shown as mean ± SEM. **(A,B,D)** *P < 0.05, **P < 0.01, and ***P < 0.001 vs. LG.

Impaired lysosomal Ca^2+^ homeostasis could lead to lysosomal dysfunction (30), and chronic exposure of HG to macrophages was demonstrated to induce the inhibition of lysosomal function (31); however, whether lysosomal Ca^2+^ homeostasis was altered under HG condition is still unclear. To examine the role of lysosomes in hyperglycemic environment in human monocytic cells, we first measured Ca^2+^ release from the lysosomes. GPN is a cathepsin C substrate that was reported to induce lysosomal Ca^2+^ release in monocytes (25). In Fluo*-*4*-*loaded U937 cells, treatment with HG (10, 20, 30 mM glucose for 48 h) or 30 mM glucose for 24, 48, or 72 h significantly reduced GPN-evoked Ca^2+^ release (Figures 1A–C), compared to low glucose (LG; 5.5 mM glucose) and 30 mM mannitol (Ma). Ma was used to as an osmotic control. Since 30 mM glucose treatment for 48 h, but not Ma, induced approximately 85% reduction of GPN-evoked Ca^2+^ release in U937 cells; therefore, it was regarded as our HG model in this study. Moreover, we also used another human monocytic cell line, THP-1, to confirm this observation. Similarly, we also observed that there was a reduction of GPN-evoked Ca^2+^ release under HG condition in THP-1 cells (Figure 1D), suggesting that HG might influence lysosomal Ca^2+^ homeostasis in human monocytic cells. In THP-1 cells, pre-treatment with U18666A, a drug that was used to deplete lysosomal Ca^2+^ store, significantly blocked GPN-evoked Ca^2+^ release (Figure 1D), this confirmed that GPN-evoked Ca^2+^ release was from the lysosomes in human monocytic cells.

**Figure 2:**
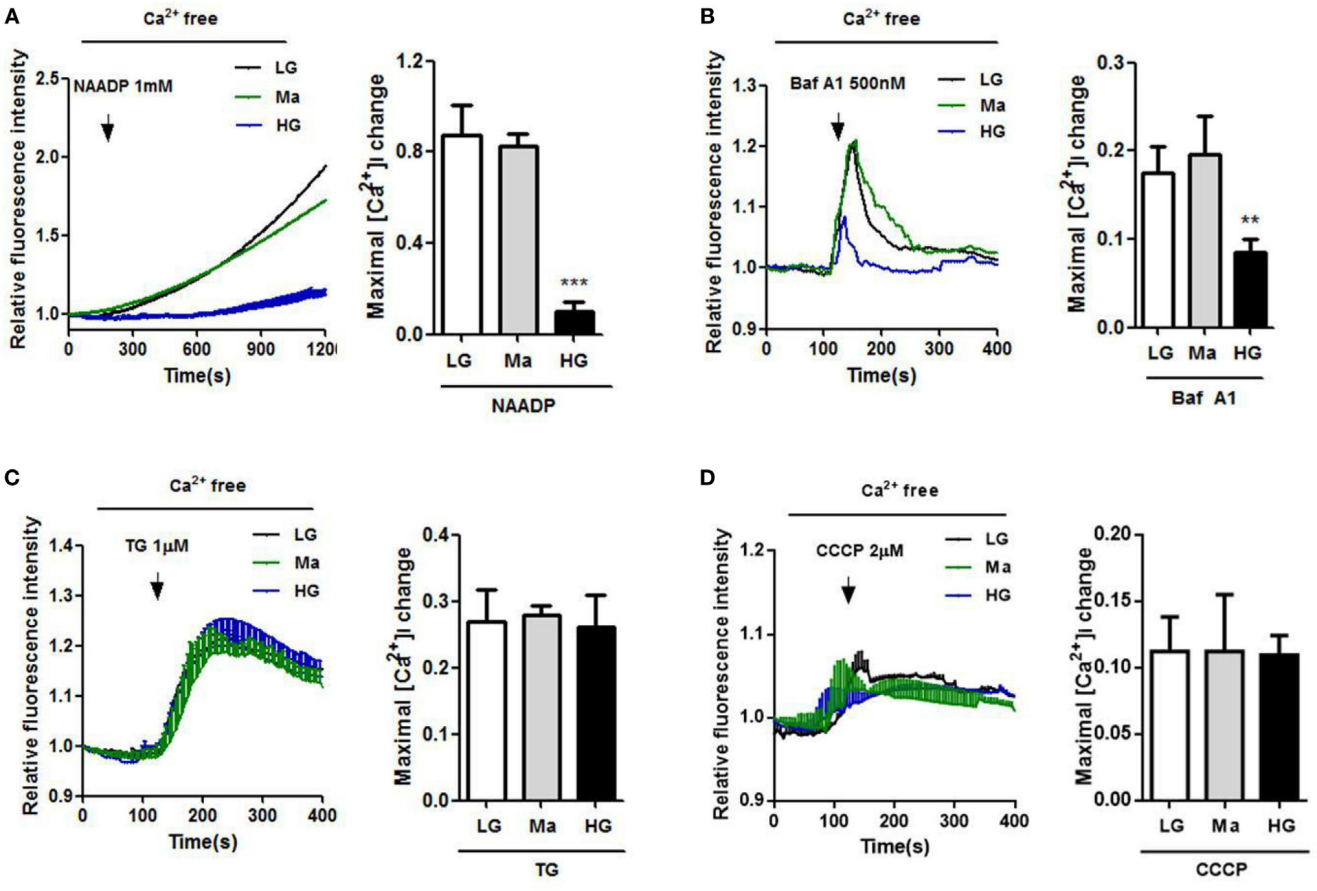
High glucose (HG) reduced Ca^2+^ release by nicotinic acid adenine dinucleotide phosphate (NAADP) and bafilomycin A1, but not by thapsigargin (TG) and carbonyl cyanide 3-chlorophenylhydrazone (CCCP) in U937 cells. **(A–D)** U937 cells were loaded with Fluo-4-AM, and were treated with indicated intracellular Ca^2+^ activators to evoke Ca^2+^ responses. Representative and relative changes in intracellular Ca^2+^ concentration ([Ca^2+^]_i_) evoked by **(A)** NAADP (1 mM), or **(B)** bafilomycin A1 (Baf A1; 500 nM), or **(C)** TG (1 µM), or **(D)** CCCP (2 µM) under low glucose (LG; 5.5 mM glucose), mannitol (Ma; 30 mM mannitol), and HG (30 mM glucose for 48 h) in U937 cells (*n* = 4–5). Data were shown as mean ± SEM. **(A–D)** **P < 0.01 and ***P < 0.001 vs. LG.

To further examine the role of Ca^2+^ homeostasis under HG condition in human monocytic cells, we used NAADP, a Ca^2+^-mobilizing secondary messenger that was known to release Ca^2+^ from the acidic endo-lysosomal vesicles (32), and bafilomycin A1, an inhibitor of the vacuolar-ATPase to induce lysosomal Ca^2+^ release. Figures 2A,B showed that NAADP- and bafilomycin A1-evoked Ca^2+^ release were significantly reduced under HG in U937 cells. By contrast, we also measured Ca^2+^ release from the ER and mitochondria under HG condition. The cells were treated with TG to release ER Ca^2+^, or with CCCP, a mitochondrial uncoupler to release mitochondria Ca^2+^. No differences in Ca^2+^ release from the ER or mitochondria were observed between LG-, Ma-, and HG-treated U937 cells (Figures 2C,D). Taken together, this suggested that HG induced a disruption of Ca^2+^ homeostasis within lysosomes, but was dispensable for ER and mitochondria Ca^2+^ in human monocytic cells.

### HG Increases Cytosolic Ca^2+^ Concentration by Reducing Lysosomal Ca^2+^ Concentration in Monocytic Cells

**Figure 3:**
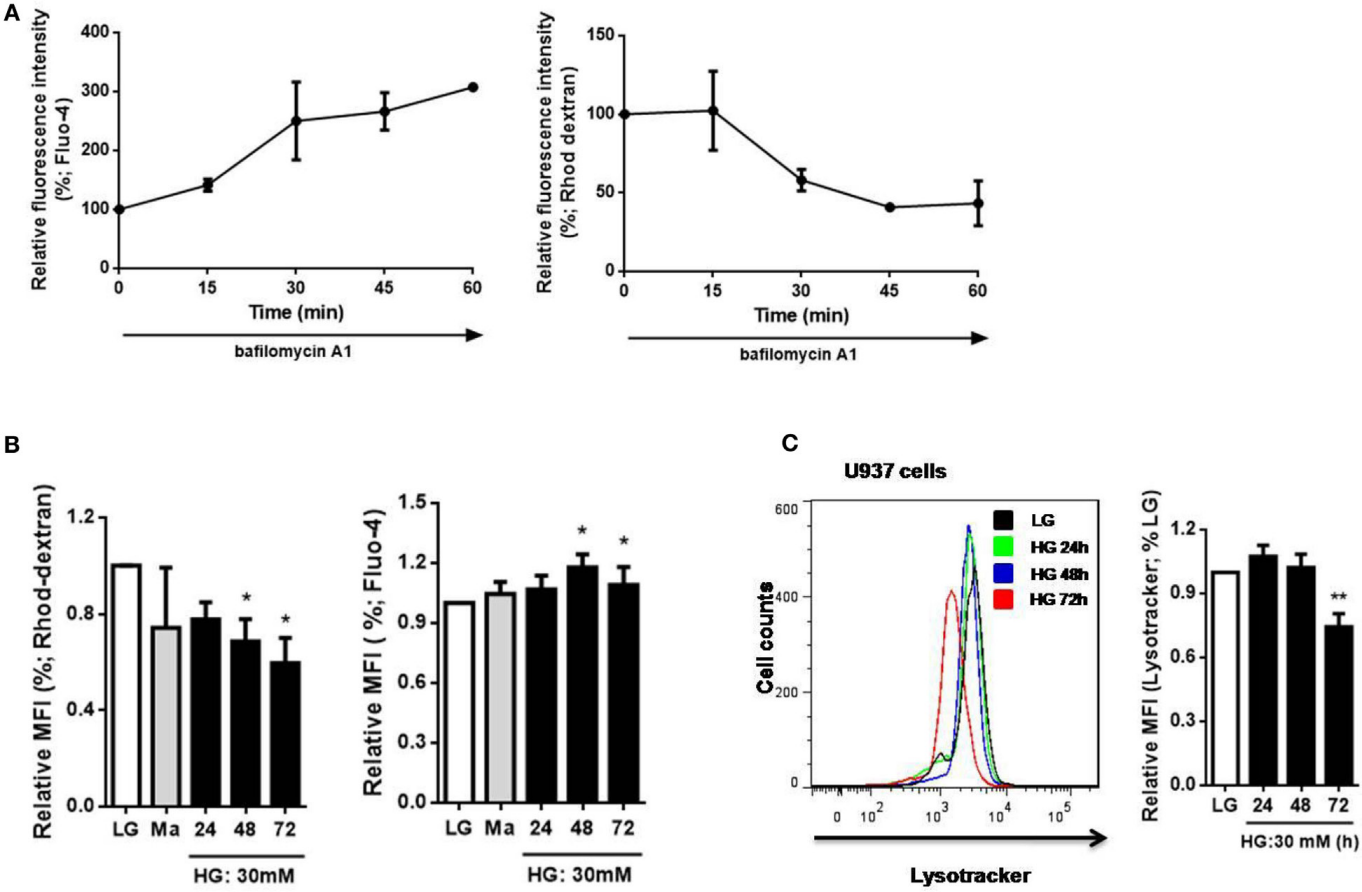
High glucose (HG) induced changes in cytosolic Ca^2+^ level by affecting lysosomal Ca^2+^ level and lysosomal function in U937 cells. **(A)** The percentages of relative median fluorescence intensity (MFI) by Fluo-4-AM or Rhod-dextran staining after bafilomycin A1 (500 nM) stimulation for 15–60 min in U937 cells. **(B,C)** The MFI by **(B)** Fluo-4 or Rhod-dextran staining, or **(C)** Lysotracker staining under low glucose (LG; 5.5 mM glucose), mannitol (Ma; 30 mM mannitol), and HG (30 mM glucose for 48 h) in U937 cells (*n* = 5). Data were shown as mean ± SEM. **(B,C)** *P < 0.05 and **P < 0.01 vs. LG.

To determine the relationship between Ca^2+^ homeostasis and lysosomes, we measured cytosolic and lysosomal Ca^2+^ levels directly with Fluo-4 and Rhod-dextran, respectively, as previously described (28). Bafilomycin A1 was reported to increase the pH level of lysosomes that increased cytosolic Ca^2+^ concentration by reducing lysosomal Ca^2+^ level (33). In agreement with that, after 60 min treatment with bafilomycin A1, an increase in Fluo-4 MFI and a decrease in Rhod-dextran MFI were observed in U937 cells (Figure 3A). This further confirmed the change in cytosolic and lysosomal Ca^2+^ levels with Fluo-4 and Rhod-dextran by bafilomycin A1. Next, we examined whether HG affected Ca^2+^ homeostasis in monocytic cells, we observed a decrease in lysosomal Ca^2+^ level with Rhod-dextran and elevation in cytosolic Ca^2+^ level with Fluo-4 under HG (30 mM; 24, 48, 72 h) in U937 cells, this strongly suggested that HG decreased lysosomal Ca^2+^ concentration and affected cytosolic Ca^2+^ homeostasis (Figure 3B). Besides, we also determined whether HG influenced lysosomal function in human monocytic cells. LysoTracker dye was used to label lysosomes in live cells (34). By using flow cytometry, we observed a significant decrease in LysoTracker staining under HG for 72 h, but not for 24 h and 48 h in U937 cells (Figure 3C), suggesting that HG for 48 h caused a defect in lysosomal Ca^2+^ store, and HG for up to 72 h could inhibit lysosomal function in human monocytic cells. Taken together, our results suggested that HG induced the loss of lysosomes, affected lysosomal and cytosolic Ca^2+^ homeostasis in human monocytic cells.

### HG Alters Intracellular Ca^2+^ Homeostasis to Mediate Lysosomal Exocytosis, Cathepsin D Activity, and IL-1β Secretion in Monocytic Cells

**Figure 4:**
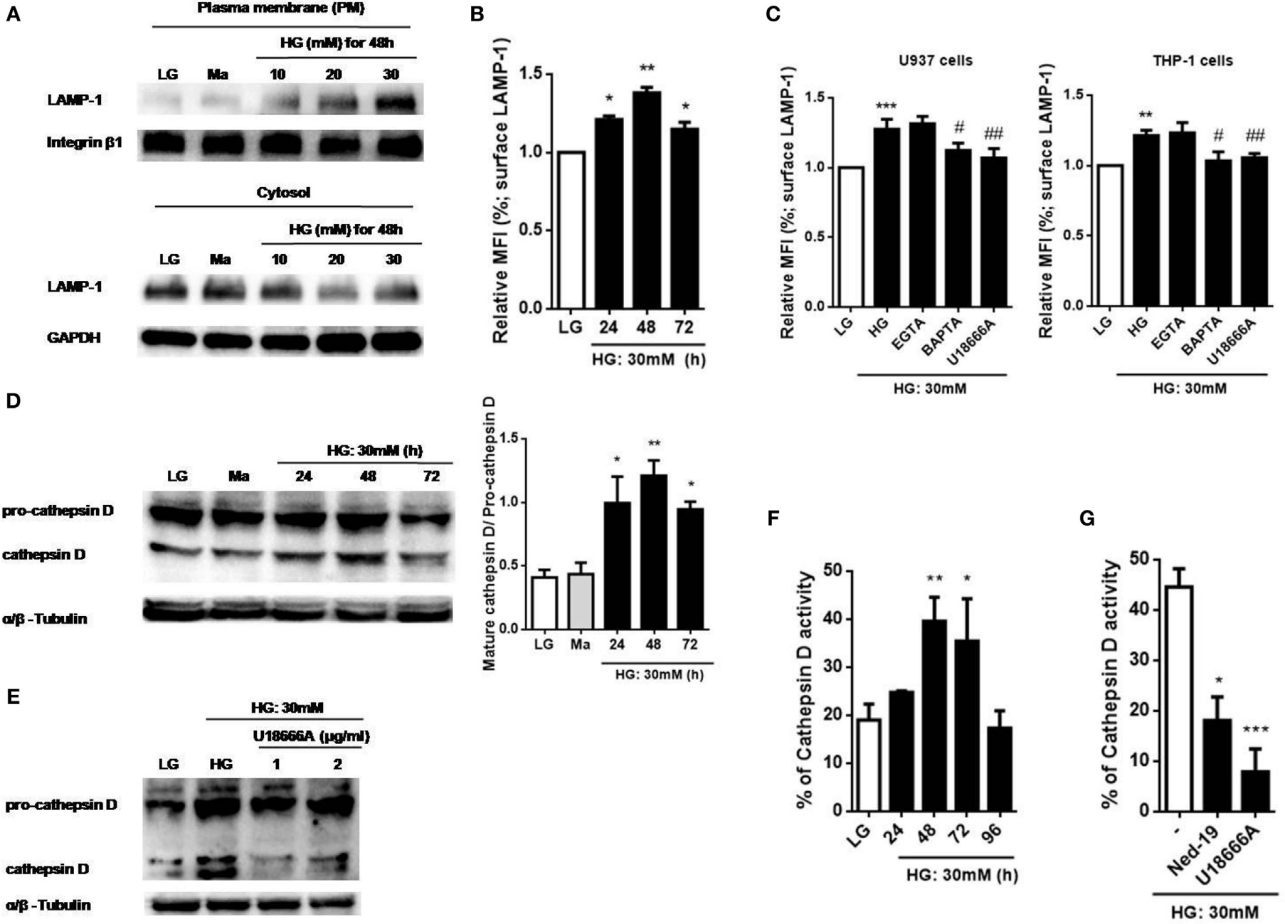
High glucose (HG) increased intracellular Ca^2+^-dependent surface lysosomal-associated membrane protein-1 (LAMP-1) level and cathepsin D maturation and activity in U937 and THP-1 cells. **(A)** Representative immunoblots for LAMP-1 and integrin β1 levels on the plasma membrane (PM), and LAMP-1 and GAPDH levels in the cytoplasm under low glucose (LG; 5.5 mM glucose), mannitol (Ma; 30 mM mannitol) and HG (10, 20, 30 mM glucose for 48 h) in U937 cells. **(B,C)** The relative median fluorescence intensity (MFI) of surface LAMP-1 staining, **(B)** under LG and HG (30 mM glucose for 24–72 h) in U937 cells, or **(C)** in the presence of ethylene glycol tetra acetic acid (EGTA) (5 mM), BAPTA (10 µM), or with the pre-treatment of U18666A (2 µg/ml) under HG (30 mM glucose for 48 h) in U937 and THP-1 cells (*n* = 4). **(D,E)** Representative immunoblots and graphs for pro- and mature cathepsin D, and α/β-Tubulin under LG or HG (30 mM glucose for 24–72 h), or **(E)** with the pre-treatment of U18666A (1, 2 µg/ml) under HG (30 mM glucose for 48 h) in U937 cells. **(F,G)** Cathepsin D activity was measured by cathepsin D activity kit. U937 cells were **(F)** stimulated with HG (30 mM glucose for 24–96 h), or **(F)** with the pre-treatment of U18666A (2 µg/ml) or *trans*-Ned-19 (Ned-19; 100 µM) under HG. The percentage of cathepsin D activity was normalized to **(F)** LG or **(G)** HG (*n* = 3). Data were shown as mean ± SEM. **(B–F)** *P < 0.05, **P < 0.01, and ***P < 0.001 vs. LG; ^#^*P* < 0.05 and ^##^*P* < 0.01 vs. HG. **(G)** *P < 0.05 and ***P < 0.001 vs. HG.

Previous studies have suggested that Ca^2+^ signals was involved in lysosomal exocytosis-mediated IL-1β secretion in response to multiple stimuli (15, 25, 35, 36), whether HG disturbed Ca^2+^ homeostasis to promote lysosomal exocytosis is still unknown. To examine lysosomal exocytosis, we stained surface LAMP-1, a marker of the lysosomal exocytosis process (37). Figure 4A showed that HG (10, 20, and 30 mM glucose for 48 h) induced LAMP-1 translocation from cytosol to the PM in a dose-dependent manner in U937 cells. Similarly, we observed that treatment with HG (30 mM glucose) for 24, 48, and 72 h significantly increased surface LAMP-1 level by flow cytometry, where it reached maximum at 48 h in U937 cells (Figure 4B), suggesting that HG induced an active movement of lysosomes toward the PM. Moreover, we also examined the effects of different Ca^2+^ chelators and blockers on the surface LAMP-1 level under HG. In U937 cells, buffering of [Ca^2+^]_i_ by BAPTA significantly inhibited HG-induced surface LAMP-1 level (Figure 4C). Besides, we observed that the depletion of lysosomal Ca^2+^ store by U18666A also blocked this effect (Figure 4C). Similar results were obtained in THP-1 cells (Figure 4C). By contrast, EGTA did not affect the LAMP-1 level (Figure 4C), suggesting that HG rapidly triggered intracellular Ca^2+^ signals, which contributed to lysosomal exocytosis in human monocytic cells. Furthermore, HG-triggered translocation of LAMP-1 was accompanied by the lysosomal hydrolase, including cathepsin D (Figure 4D). We found that HG induced the maturation and activity of cathepsin D with maximal effects occurring at 48 h in U937 cells, whereas pre-treatment with U18666A could block these effects (Figures 4D–G). Similarly, Ned-19, an inhibitor of NAADP that blocks NAADP-induced Ca^2+^ mobilization from the lysosomes, also inhibited HG-induced cathepsin D activity in U937 cells (Figure 4G). This indicated that lysosomal Ca^2+^ signals was involved in HG-induced lysosomal exocytosis.

**Figure 5:**
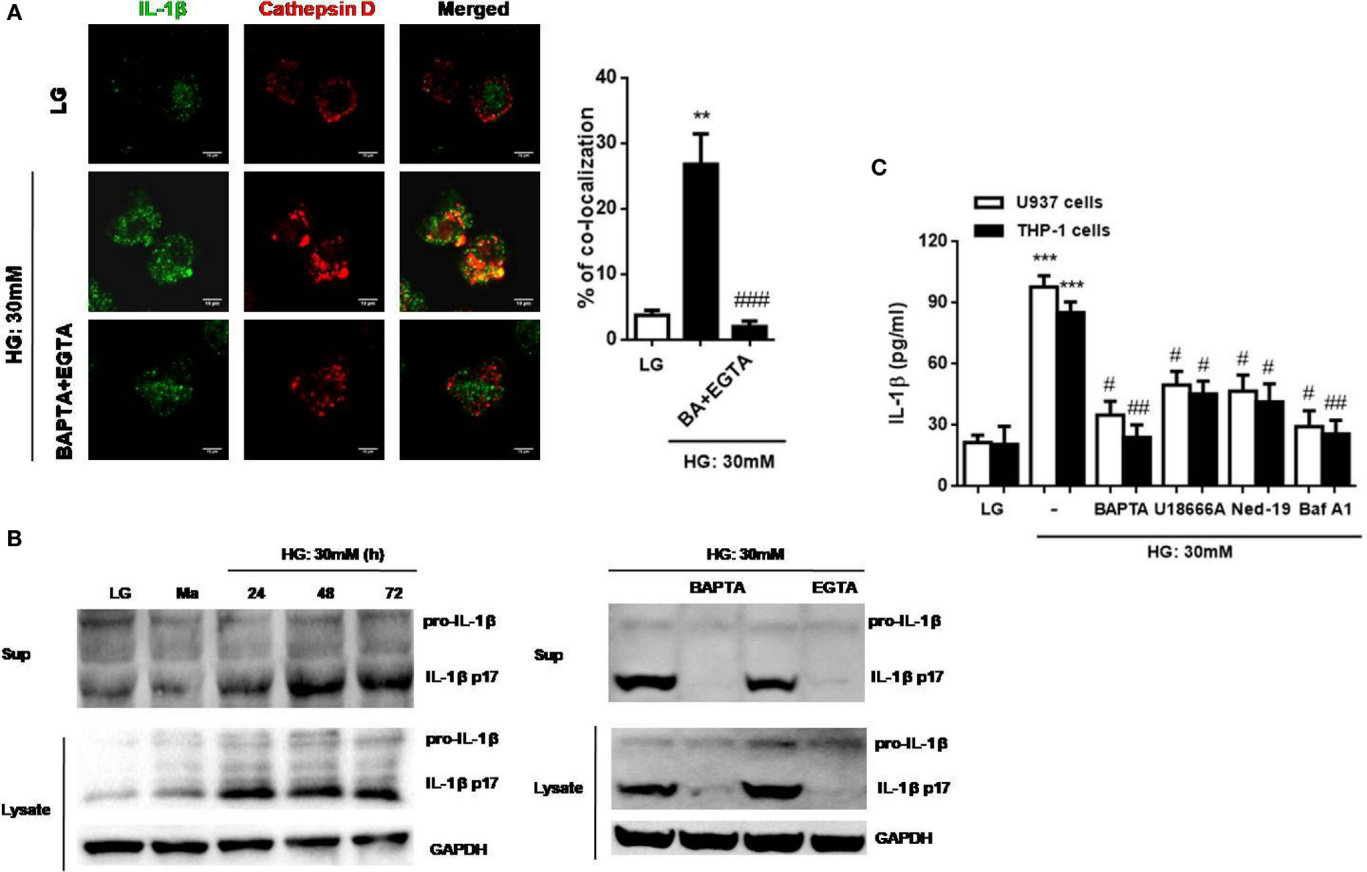
High glucose (HG) induced cathepsin d-dependent interleukin-1β (IL-1β) secretion, which was dependent on lysosomal Ca^2+^ release in U937 and THP-1 cells. **(A)** Immunofluorescence images showing the location of IL-1β and cathepsin D in fixed U937 cells under HG (30 mM glucose for 48 h) by confocal microscopy. The U937 cells were pre-treated with BAPTA (10 µM) plus ethylene glycol tetra acetic acid (EGTA) (5 mM). The percentages of co-localization were calculated as the average volume of the overlapping areas (*n* = 4). **(B)** Representative immunoblots for pro-IL-1β, IL-1β (p17), and GAPDH protein expressions under low glucose (LG; 5.5 mM glucose for 48 h) or HG (30 mM glucose for 24–72 h), or in the presence of BAPTA (BA; 10 µM) or EGTA (5 mM) under HG (30 mM glucose for 48 h) in U937 cells. **(C)** ELISA for IL-1β secretion from the supernatants of treated cells. U937 cells were stimulated with HG (30 mM glucose for 48 h) in the presence of BAPTA (10 µM), or with the pre-treatment of U18666A (2 µg/ml), *trans*-Ned-19 (Ned-19; 100 µM), or bafilomycin A1 (Baf A1; 500 nM). Data were shown as mean ± SEM. **(A,C)** **P < 0.01 and ***P < 0.001 vs. LG; ^#^*P* < 0.05, ^##^*P* < 0.01, and ^###^*P* < 0.001 vs. HG.

We then investigated whether IL-1β was accompanied by lysosomal exocytosis. The intracellular distribution of IL-1β and cathepsin D was examined under HG in U937 cells by confocal microscopy. We found out that IL-1β was co-localized with cathepsin D under HG, whereas this effect was abolished by the removal of Ca^2+^ with BAPTA plus EGTA (Figure 5A). Moreover, during HG (30 mM glucose) stimulation, IL-1β maturation and release were also abolished by buffering of [Ca^2+^]_i_ with BAPTA and the removal of extracellular Ca^2+^ with EGTA in U937 cells (Figure 5B). Meanwhile, we also examined the effect of Ca^2+^ chelators and agents on IL-1β secretion under HG condition in U937 and THP-1 cells. As expected, BAPTA significantly reduced IL-1β secretion by HG (Figure 5C). To further investigate whether lysosomal Ca^2+^ release participated in IL-1β secretion by HG, we used three antagonists, U18666A, Ned-19, and bafilomycin A1. Figure 5C showed that U18666A, Ned-19, and bafilomycin A1 markedly blocked HG-induced IL-1β secretion in U937 and THP-1 cells. Taken together, these results indicated that HG altered lysosomal Ca^2+^ homeostasis, which resulted in an increase in [Ca^2+^]_i_ and surface LAMP-1 level, facilitation in lysosomal exocytosis, lysosomal cathepsin D maturation and activity, and IL-1β release in human monocytic cells.

### HG Induces Lysosomal Ca^2+^ Release-Dependent TFEB Translocation in Monocytic Cells

**Figure 6:**
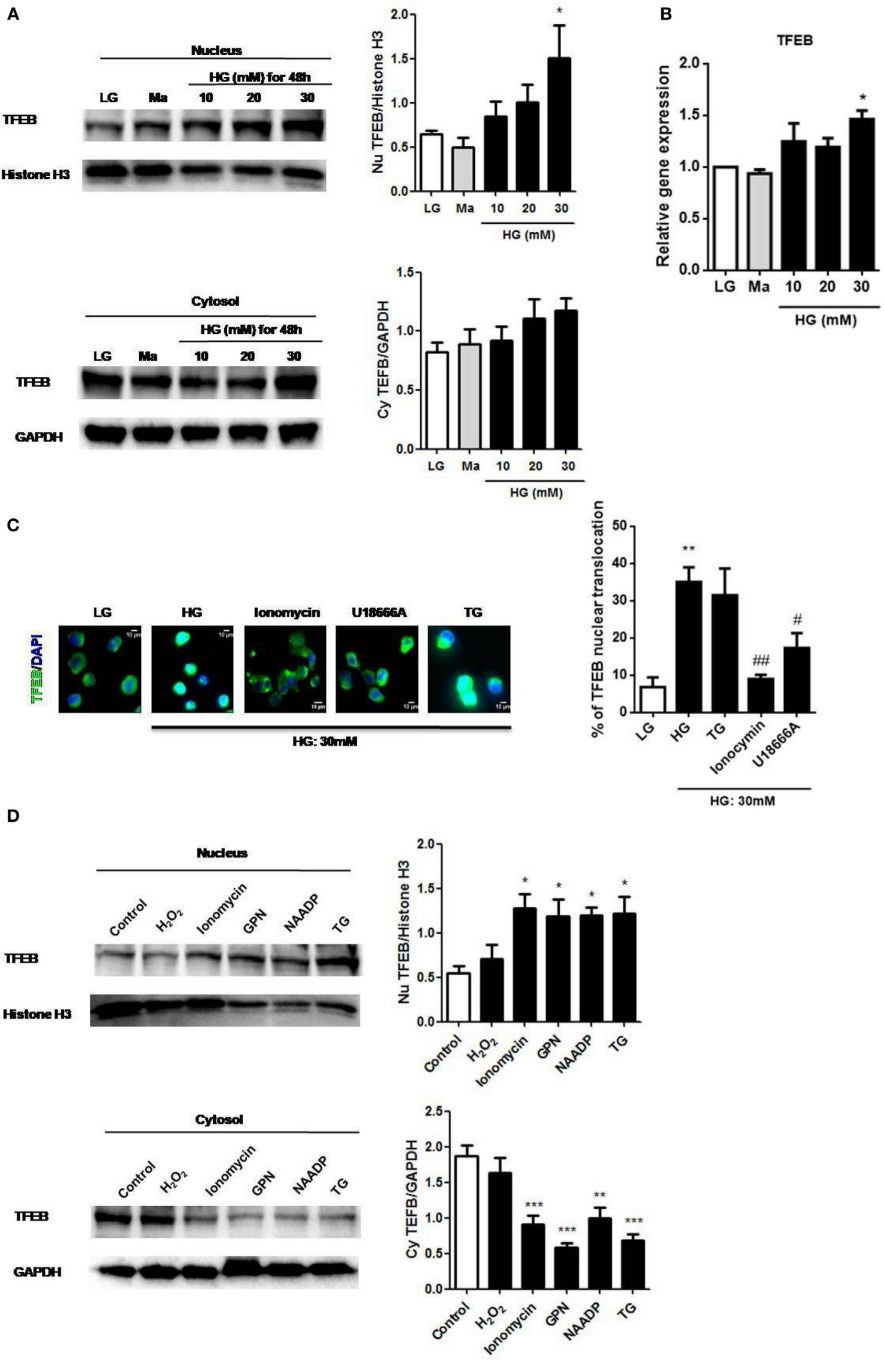
High glucose (HG) upregulated transcription factor EB (TFEB) expression and lysosomal Ca^2+^-dependent TFEB nuclear translocation in U937 cells. **(A)** Representative immunoblots and graphs for TFEB and Histone H3 expressions in the nucleus, and TFEB and GAPDH expressions in the cytoplasm under low glucose (LG; 5.5 mM glucose), mannitol (Ma; 30 mM), or HG (10, 20, 30 mM glucose for 48 h) in U937 cells. The relative expression of TFEB was normalized to representative controls (Histone H3/GAPDH) (*n* = 4). **(B)** Relative gene expression of TFEB under LG, Ma, or HG (10, 20, and 30 mM glucose for 48 h) in U937 cells (*n* = 5). **(C)** Immunofluorescence images and representative graph showing the nuclear translocation of TFEB in U937 cells that were pre-treated with ionomycin (10 µM), U18666A (2 µg/ml), or thapsigargin (TG; 1 µM) under HG (30 mM glucose for 48 h). The graph represented the percentage of the cells with nuclear translocation of TFEB (*n* = 4). **(D)** Representative immunoblots and graphs for TFEB and Histone H3 expressions in the nucleus, and TFEB and GAPDH expressions in the cytoplasm after stimulation with H_2_O_2_ (400 µM), ionomycin (1 µM), glycyl-l-phenylalanine-beta-naphthylamide (GPN; 400 µM), nicotinic acid adenine dinucleotide phosphate (NAADP; 1 mM), or TG (400 nM) in U937 cells. The relative protein expression of TFEB was normalized to representative controls (histone H3/GAPDH) (*n* = 4). Data were shown as mean ± SEM. **(A–C)** *P < 0.05 and **P < 0.01 vs. LG; ^#^*P* < 0.05 and ^##^*P* < 0.01 vs. HG. **(D)** *P < 0.05, **P < 0.01, and ***P < 0.001 vs. control.

The activation of TFEB was reported to regulate lysosomal exocytosis by raising [Ca^2+^]_i_ (22); therefore, we examined whether it was also involved in HG stimulation. Immunoblotting results showed that HG increased TFEB translocation to the nucleus in a dose-dependent manner in U937 cells (Figure 6A). In addition to the nuclear translocation of TFEB, we also observed that HG upregulated TFEB mRNA in U937 cells (Figure 6B), indicating that HG did not only induce TFEB activation, but could also increase its mRNA expression. Notably, we found that the depletion of internal Ca^2+^ stores by ionomycin (28), or U18666A significantly reduced HG-induced nuclear translocation of TFEB (Figure 6C). By contrast, the depletion of ER Ca^2+^ store by TG had no effect on it (Figure 6C), suggesting that Ca^2+^ release from the lysosomes, but not from the ER, mediated the activation of TFEB under HG. Conversely, short and acute exposure to ionomycin, GPN, NAADP, or TG, that triggered internal Ca^2+^ release, could significantly induce nuclear translocation of TFEB in U937 cells (Figure 6D). However, H_2_O_2_ stimulation, which was reported to regulate monocytic function *via* extracellular Ca^2+^ influx (38), did not induce nuclear translocation of TFEB (Figure 6D). Therefore, our results supported that lysosmal Ca^2+^ signals played a key role in the regulation of TFEB translocation during HG condition in human monocytic cells.

### TFEB Regulates HG-Induced Lysosomal Exocytosis and Pro-IL-1β Synthesis to Mediate IL-1β Secretion in Monocytic Cells

**Figure 7:**
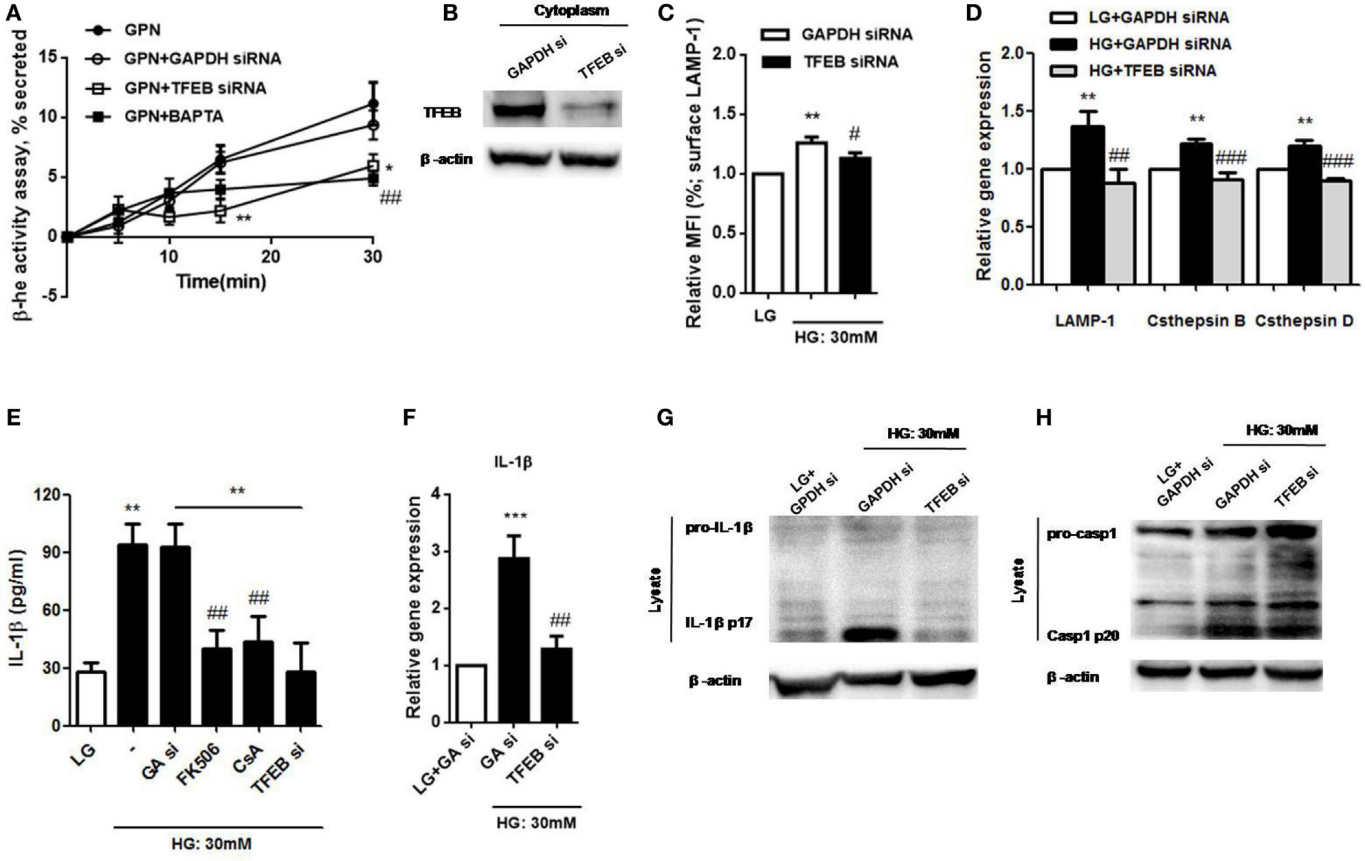
High glucose (HG) induced lysosomal Ca^2+^-dependent lysosomal exocytosis and interleukin-1β (IL-1β) secretion *via* calcineurin/transcription factor EB (TFEB) pathway in U937 cells. **(A)** The percentage of β-hexosaminidase activity was induced by glycyl-l-phenylalanine-b-napthylamide (GPN). U937 cells were treated with GPN (400 µM) in the presence of GAPDH small interfering RNA (siRNA) or TFEB siRNA or BAPTA (10 µM) (*n* = 4). The results were normalized to control. **(B)** Representative immunoblots for TFEB and β-actin in GAPDH siRNA (GAPDH si)- or TFEB siRNA-treated cells (TFEB si) (*n* = 3). **(C)** The relative median fluorescence intensity (MFI) of surface lysosomal-associated membrane protein-1 (LAMP-1) staining that were in the presence of GAPDH or TFEB siRNA under low glucose (LG; 5.5 mM glucose) or HG (30 mM glucose for 48 h) (*n* = 4). **(D)** The relative gene expressions of LAMP-1, cathepsin D, and cathepsin B in the presence of GAPDH or TFEB siRNA under LG or HG (30 mM glucose for 48 h) in U937 cells (*n* = 5). **(E)** ELISA for IL-1β secretion from the supernatants of U937 cells that were pre-treated with FK506 (25 µM) or cyclosporin A (Cs A; 10 µM), or in the presence of GAPDH siRNA (GA si) or TFEB siRNA (TFEB si) under LG or HG. **(F)** The relative gene expressions of IL-1β in the presence of GAPDH or TFEB siRNA under HG in U937 cells (*n* = 4). **(G,H)** Representative immunoblots for pro-IL-1β, IL-1β p17, or pro-caspase-1, cleaved caspase-1 (p20) and β-actin in the presence of GAPDH or TFEB siRNA under LG or HG (30 mM glucose for 48 h) in U937 cells (*n* = 4). Data were shown as mean ± SEM. **(A)** *P < 0.05 and **P < 0.01 vs. GPN + GAPDH siRNA; ^##^*P* < 0.01 vs. GPN. **(C,E)** **P < 0.01 vs. LG; ^#^*P* < 0.05 and ^##^*P* < 0.01 vs. HG. **(D,F)** **P < 0.01 and ***P < 0.001 vs. LG + GAPDH siRNA; ^##^*P* < 0.01 and ^###^*P* < 0.001 vs. HG + GAPDH siRNA.

We next investigated whether TFEB could regulate lysosomal exocytosis in U937 cells. Lysosomal Ca^2+^ response induced by GPN was proposed to be responsible for lysosomal exocytosis in human monocytes (25). Here, we measured the release of the lysosomal marker enzyme, β-hexosaminidase, to examine lysosome exocytosis. Our results demonstrated that GPN-induced β-hexosaminidase release in a time-dependent manner, and it was inhibited by TFEB siRNA and BAPTA in U937 cells (Figure 7A); this suggested that intracellular Ca^2+^ signals was involved in GPN-induced lysosomal exocytosis through TFEB pathway. The efficiency of the knockdown was shown by immunoblotting (Figure 7B). Moreover, we also found that HG-induced surface LAMP-1 level was reduced by TFEB siRNA (Figure 7C). Next, to further determine whether TFEB could control lysosomal exocytosis through its target genes, we measured the mRNA levels of TFEB target genes that were previously linked to lysosomal exocytosis, including LAMP-1, cathepsin B, and cathepsin D (15, 29, 39). We demonstrated that the mRNA expressions of LAMP-1, cathepsin B, and cathepsin D were upregulated under HG in U937 cells (Figure 7D). As expected, these effects were abolished by TFEB siRNA (Figure 7D), suggesting that TFEB directly controlled lysosomal exocytosis under HG condition. In addition, previous study demonstrated that calcineurin interacted with TFEB and modulated its activation (24). We observed that calcineurin inhibitors, cyclosporin A and FK506, significantly inhibited HG-induced IL-1β secretion in U937 cells (Figure 7E); so this further confirmed that HG induced lysosomal exocytosis-mediated IL-β secretion *via* calcineurin/TFEB pathway. Besides, our results also found that HG-mediated upregulation of IL-1β mRNA level and its maturation were suppressed by TFEB siRNA in U937 cells (Figures 7F,G). By contrast, TFEB siRNA did not induce HG-induced caspase-1 cleavage (p20) (Figure 7H). Taken together, this further suggested that TFEB/calcineurin pathway was responsible for HG-induced IL-1β release *via* regulation of synthesis of pro-IL-1β and lysosomal exocytosis, but independent of caspase-1 activation in human monocytic cells.

## Discussion

Interleukin-1β, an inducer of various pro-inflammatory cytokines and chemokines, was implicated in driving tissue inflammation during T2DM (40, 41), and was tightly associated with promoting β-cell death, impaired insulin sensitivity and enhancing the adhesion capacity of circulating monocytes to the vascular endothelium (42–44). Recent studies demonstrated that targeting IL-1β, but not TNF-α antagonism, had beneficial effects for treating T2DM and its complications (3, 45–47). The present study provided mechanistic insights into IL-1β release induced by HG, which was mediated by lysosomal exocytosis *via* TFEB/calcineurin pathway in human monocytic cell lines, U937 and THP-1 cells. Furthermore, our results demonstrated that HG could cause a defect in lysosomal Ca^2+^ store and altered cytosolic Ca^2+^ homeostasis, which was essential for lysosomal exocytosis.

Interleukin-1β is one of the major inflammatory cytokines that is critical for chronic inflammatory response during metabolic disorders, including obesity and T2DM. The secretion of IL-1β is primarily from monocytes and macrophages (11), and HG, a characteristic of T2DM, could upregulate IL-1β mRNA and stimulate its secretion in human monocytes, contributing to impaired insulin secretion and signaling (48, 49). Indeed, there are several steps for IL-1β secretion, first is to produce inactive precursor, pro-IL-1β, which is then cleaved by caspase–1 to produce mature IL-1β, and the maturation of IL-1β should be secreted through non-conventional secreting pathway (50). Our previous study has demonstrated that HG induced NLRP3 inflammasome and caspase-1 activation, which contributed to IL-1β processing and secretion in monocytes (10); however, the mechanisms of secreting IL-1β into extracellular milieu are unclear. In human monocytes, the exocytosis of secretory lysosomes was a key mechanism for IL-1β secretion, and this required the elevation of [Ca^2+^]_i_ and Ca^2+^-dependent phospholipases (15, 41). Our results also showed that HG significantly increased [Ca^2+^]_i_ by reducing lysosomal Ca^2+^ level, and HG only affected lysosomal Ca^2+^ homeostasis but not ER and mitochondria Ca^2+^ homeostasis in human monocytic cells. It has been suggested that lysosomal Ca^2+^ signals could be linked to regulating endolysosome function, including altering lysosomal morphology, maintaining cytosolic Ca^2+^ homeostasis and lysosomal exocytosis (24, 25, 33, 51). We found out that lysosomal Ca^2+^ is a critical determinant of maintaining intracellular Ca^2+^ homeostasis under HG condition. HG raised [Ca^2+^]_i_ that was originated from the lysosomes, and this lysosomal Ca^2+^ signals enhanced lysosomal exocytosis markers, like surface level of LAMP, cathepsin D, and β-hexosaminidase activity, which were critical for lysosome trafficking to the PM (lysosomal exocytosis). Therefore, this lysosomal Ca^2+^ contributed to secreting IL-1β into extracellular milieu in human monocytic cells.

Transcription factor EB was shown to regulate lysosomal exocytosis (22, 52). Recent study demonstrated that lysosomal stresses, such as Ox-LDL and cholesterol crystals, could induce TFEB nuclear translocation and the activation of lysosomal and autophagy genes in macrophages (53). Here, we showed that HG upregulated TFEB expression and induced TFEB nuclear translocation in U937 monocytic cells, and which was dependent on intracellular Ca^2+^, particularly lysosomal Ca^2+^. Interestingly, our results demonstrated that several internal Ca^2+^ activators, such as ionomycin, GPN, NAADP, and TG, were capable of inducing TFEB nuclear translocation. Therefore, it was likely that HG induced TFEB activation as a consequence of Ca^2+^ release from the lysosomes. Moreover, in other various cells, such as fibroblasts, neuronal cells, and osteoclasts, it was reported that overexpression of TFEB could mediate lysosomal exocytosis by raising [Ca^2+^]_i_ (22, 54). Similarly, our results showed that TFEB was critical for HG-induced upregulation of lysosomal gene expressions, such as cathepsin D and LAMP-1, in U937 monocytic cells. Therefore, it was not surprising that TFEB could regulate Ca^2+^-dependent lysosomal exocytosis *via* lysosomal genes under HG condition. Although our study with other study showed that lysosomal exocytosis was regulated by TFEB (22), a direct regulation of IL-1β secretion by TFEB was not studied. As expected, we found that HG induced lysosomal exocytosis through calcineurin/TFEB pathway. We further studied the link between TFEB and IL-1β secretion, our results observed that TFEB significantly suppressed mRNA level of IL-1β, but it was dispensable for caspase-1 cleavage under HG. This suggested that TFEB play a critical role for regulating lysosomal exocytosis and pro-IL-1β synthesis, but not participate in caspase-1-dependent processing of pro-IL-1β into mature IL-1β. In addition, the inhibition of calcineurin, a binding partner of TFEB and mediates its activation (24), was reported to reduce IL-1β secretion *via* the inhibition of pro-IL-1β levels during lipotoxic inflammasome activation (55); this further supported our study, which suggested that calcineurin/TFEB activation was involved in the upregulation of IL-1β level, and subsequently affected its secretion. Taken together, our results suggested that lysosomal Ca^2+^-mediated TFEB activation could control lysosomal exocytosis through LAMP-1 and cathepsin D, and regulate intracellular pro-IL-1β synthesis by HG in human monocytic cells.

Regarding to the function of lysosomes, prolonged HG treatment was shown to inhibit lysosomal function in different cell types (31, 56–58). We showed that HG for 72 h, but not 48 h, resulted in the loss of lysosomes; however, a defect in lysosomal Ca^2+^ store was started to occur at 48 h, which suggested that lysosomal Ca^2+^ depletion was an early event of lysosomal disruption. Since impaired lysosomal Ca^2+^ store was suggested to induce lysosomal dysfunction (30, 59), we also observed that HG induced a decrease in lysosomal Ca^2+^ level and an increase in intracellular Ca^2+^ level. This observation suggested that HG might induce lysosomal Ca^2+^ release to raise cytosolic Ca^2+^ concentration and lead to disruption of lysosomal function by preventing Ca^2+^ refilling back to lysosomes. In particular, ER Ca^2+^ store and lysosomal pH gradient were responsible for driving Ca^2+^ refilling of lysosomes (30, 33). Our results demonstrated that HG induced an increase in cytosolic Ca^2+^ level, a defect in lysosomal Ca^2+^ level, but did not affect ER Ca^2+^ store. Therefore, it was likely that HG induced aberrant lysosomal pH, which contributed to the increase in [Ca^2+^]_i_ and impaired lysosomal Ca^2+^ store, as supported by two studies (31, 33). Besides, exposure to HG more than 48 h (~72 h) prevented Ca^2+^ refilling of lysosomes, and lysosomal exocytosis, which was accompanied with enhanced cathepsin D activity, reaching maximum at HG for 48 h and then decreased after 72 h in monocytic cells. These observations suggested that HG induced the processing of exocytosis must be under normal lysosomal function (exposure to HG less than 48 h) to allow lysosomal Ca^2+^ release under physiological level.

In our previous work, we identified some novel mechanisms involved in the activation of NLRP3 inflammasome under HG in human monocytic cells. We demonstrated that TRPM2-mediated Ca^2+^ influx could contribute to HG-induced ROS overproduction and NLRP3 inflammasome activation, leading to IL-1β maturation and release (10). Notably, several studies suggested that Ca^2+^ signals was critical for IL-1β secretion induced by variety of stimulus, which was not only mediated through NLRP3 inflammasome activation, but also by lysosomal exocytosis (25, 35, 60, 61). In this study, we demonstrated two important pathways of HG-induced IL-1β secretion. First, lysosomal Ca^2+^ release played a vital role in HG-induced secreting IL-1β into extracellular milieu *via* lysosomal exocytosis/TFEB pathway. Second, TFEB could promote pro-IL-1β synthesis induced by HG. Taken together, our previous and the present study suggested that TRPM2-mediated Ca^2+^ influx regulate NLRP3 inflammasome activation, whereas internal Ca^2+^, particularly lysosomal Ca^2+^ release, was associated with triggering TFEB activation, which contributed to pro-IL-1β synthesis and secretion. Moreover, lysosomal Ca^2+^ signals was also responsible for secreting IL-1β into extracellular milieu *via* lysosomal exocytosis in human monocytic cells. The important role of IL-1β in T2DM has been recognized in the recent years (2, 3), and this observation provided more insight into mechanisms of IL-1β secretion in T2DM.

In conclusion, we demonstrated that HG could alter intracellular Ca^2+^ homeostasis, particularly lysosomal Ca^2+^ homeostasis, to trigger the activation of calcineurin and TFEB, a master gene for lysosomal function, in monocytic cells. Hence, TFEB could modulate lysosomal exocytosis by enhancing [Ca^2+^]_i_ and contributed to secreting IL-1β into extracellular milieu under HG. Our results also demonstrated that lysosomal Ca^2+^ release by GPN or NAADP was sufficient for TFEB activation and induction of lysosomal exocytosis, suggesting that lysosomal Ca^2+^ signals was crucial for lysosomal exocytosis-dependent IL-1β release in monocytic cells. These findings provided an understanding of the underlying mechanisms of secreting IL-1β into extracellular milieu by HG, with a focus on the involvement of lysosomal Ca^2+^ signals in lysosomal exocytosis in monocytic cells.

## Author Contributions

YK, SL, and MH conceived and designed the study; HT and CV performed the experiments; HT and MH drafted the manuscript.

## Conflict of Interest Statement

The authors declare that the research was conducted in the absence of any commercial or financial relationships that could be construed as a potential conflict of interest.
